# Internal medicine board certification and career pathways in Japan

**DOI:** 10.1186/s12909-017-0919-y

**Published:** 2017-05-08

**Authors:** Soichi Koike, Masatoshi Matsumoto, Hiroo Ide, Hideaki Kawaguchi, Masahisa Shimpo, Hideo Yasunaga

**Affiliations:** 1 Division of Health Policy and Management, Center for Community Medicine, Jichi Medical University, 3311-1 Yakushiji, Shimotsuke, Tochigi 329-0498 Japan; 2 Department of Health Management and Policy, Graduate School of Medicine, The University of Tokyo, 7-3-1 Hongo, Bunkyo, Tokyo 113-0033 Japan; 3 Department of Community Based Medical System, Institute of Biomedical and Health Sciences, Hiroshima University, 1-2-3 Kasumi, Minami-ku, Hiroshima 734-8551 Japan; 4 Department of Medical Community Network and Discharge, Chiba University Hospital, 1-8-1 Inohana, Chuo, Chiba 260-8677 Japan; 5 Department of Biomedical Informatics, The University of Tokyo, 7-3-1 Hongo, Bunkyo, Tokyo 113-0033 Japan; 6 Division of Cardiovascular Medicine, Department of Medicine, Jichi Medical University School of Medicine, 3311-1 Yakushiji, Shimotsuke, Tochigi 329-0498 Japan; 7 Department of Clinical Epidemiology and Health Economics, School of Public Health, The University of Tokyo, 7-3-1 Hongo, Bunkyo, Tokyo 113-0033 Japan

**Keywords:** Board certification, Subspecialty, Maintenance of certification, Career pathway, Internal medicine

## Abstract

**Background:**

Establishing and managing a board certification system is a common concern for many countries. In Japan, the board certification system is under revision. The purpose of this study was to describe present status of internal medicine specialist board certification, to identify factors associated with maintenance of board certification and to investigate changes in area of practice when physicians move from hospital to clinic practice.

**Methods:**

We analyzed 2010 and 2012 data from the Survey of Physicians, Dentists and Pharmacists. We conducted logistic regression analysis to identify factors associated with the maintenance of board certification between 2010 and 2012. We also analyzed data on career transition from hospitals to clinics for hospital physicians with board certification.

**Results:**

It was common for physicians seeking board certification to do so in their early career. The odds of maintaining board certification were lower in women and those working in locations other than academic hospitals, and higher in physicians with subspecialty practice areas. Among hospital physicians with board certification who moved to clinics between 2010 and 2012, 95.8% remained in internal medicine or its subspecialty areas and 87.7% maintained board certification but changed their practice from a subspecialty area to more general internal medicine.

**Conclusion:**

Revisions of the internal medicine board certification system must consider different physician career pathways including mid-career moves while maintaining certification quality. This will help to secure an adequate number and distribution of specialists. To meet the increasing demand for generalist physicians, it is important to design programs to train specialists in general practice.

## Background

Establishing and maintaining a quality assurance system for medical practitioners is a priority in the postgraduate and continuing education systems. The establishment and management of a board certification system is a common concern for many countries.

Different countries have different certification and maintenance systems based on their specific healthcare delivery systems. Common issues are the quality of care [1–3], requirements and conditions of specialty certification, curriculum for board certification, maintenance of certification and demand for specialists [4–11]. A United States study of medical school graduates from the 1997–2000 cohort found that 87.3% had American Board of Medical Specialties certification and argued that board certification was emerging as a de facto requirement for full participation of medical practitioners in the US healthcare system [12].

In Japan, the board certification system has developed and has operated by respective academic societies; it is not directly linked to reimbursement systems and it is not mandatory hospitals and clinics to be staffed by board certified medical practitioners. The number of medical practitioners with board certification has been increasing. The Ministry of Health, Labour and Welfare reported that 56.9% of medical practitioners had one or more board certifications in 2014 [13], a 2.9% increase from 2012 [14]. The Commission on the Reform of the Board Certified System recommended that awareness be raised of the need for standardization that a quality assurance system of board certification be established and that maldistribution of board certified medical practitioners be addressed. It was also recommended that an independent organization for certification of specialists and training programs be established. A two-tier board system for basic and subspecialty board certification has been proposed, with basic certification mandatory before subspecialty board certification. It was also recommended to establish general practice as a basic area of board certification [15].

It was planned that the new system would be implemented from 2017; however, relevant stakeholders have not yet reached agreement and implementation has been postponed. In December 2016, the Japanese Medical Specialty Board released its Guidelines for Board Certification. It requires at least 5 years of training (including 2 years of postgraduate clinical training) after medical practitioner registration and successful completion of the training program for basic board certification. The duration of certification is 5 years, and is renewable if the requirements for maintenance of board certification are met [16].

The internal medicine board certification system was first introduced in 1968 as Fellow/Board Certified Membership of the Japanese Society of Internal Medicine. It required physicians to complete 5 years of training at accredited educational facilities and pass and examination. The system for internal medicine subspecialty board certification followed; there are now 13 subspecialties in internal medicine [17]. In 1994 “board-certified specialists in internal medicine” became known as “certified physicians and board-certified specialists in general internal medicine” [18]. Concurrently with the government commission’s recommendations, the Japanese Society of Internal Medicine revised the quantity of training required to achieve specialist board certification by requiring 3 years of training in internal medicine after 2 years of postgraduate clinical training had been completed, increased from a period of 3 years of training that included 2 years of postgraduate clinical training to become Board Certified Member of the Society.

Analysis of the physician career pathway can inform the design of human resources systems. Japan has an aging population that will require greater availability of family and general medicine. The demand for board certification will also increase. The internal medicine career pathway will change under these circumstances. There have been several studies of the Japanese board certification systems in surgery [19, 20], cerebrovascular surgery [21], anesthesiology [22], head and neck surgery [23], and consultation-liaison psychiatry [24]. In our previous study of surgeons, we found that the odds of women maintaining board certification was lower, even after adjusting for other factors. As the proportion of Japanese physicians is increasing, there is increasing interest in career differences between men and women in internal medicine [20].

The purpose of this study was to investigate the current status of board certification in internal medicine, identify factors associated with the maintenance of board certification, and to analyze change of practice area when internal medicine specialists left hospitals to practice in clinics. Finally, we also aimed to discuss the potential policy implications of our findings.

## Methods

To examine the current status and dynamics of board certification in internal medicine, factors associated with maintenance of board certification, and the career transitions of hospital-based specialists, we merged the data of the 2010 and 2012 Survey of Physicians, Dentists and Pharmacists. The Survey of Physicians, Dentists and Pharmacists is a national census survey of physicians conducted every 2 years by the Ministry of Health, Labour and Welfare. In Japan, all licensed medical practitioners are required to report their working status in the biannual Survey of Physicians, Dentists and Pharmacists. Although the survey was designed as a census, the estimated reporting rate for the 2000 survey was 90.3% [25]. The survey collects the following self-reported snapshot data about each physician on December 31: sex; date of birth; place of work; type of working facilities; area of practice and board certification status. Survey form is available from the Ministry’s website [26]. We obtained permission from the Ministry to analyze the survey data.

**Fig. 1:**
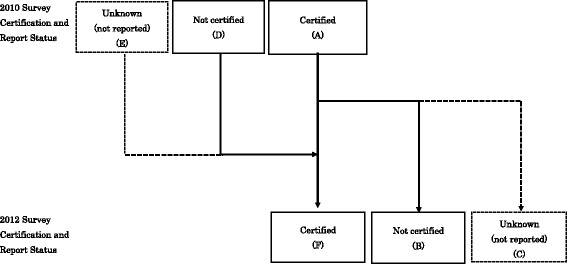
The change in certification status between 2010 and 2012 by physician registration year. Physicians who reported they were board certified in 2010 (*A*) either kept certification (*F*), lost certification (*B*) or their status became unknown (not reported) (*C*) in 2012. In addition to physicians certified both in 2010 and 2012, some were not certified in 2010 but certified in 2012 (*D*) and some whose certification status was known (not reported) in 2010 appeared to be certified in 2012 (*E*)

To examine the dynamics of board certification, we analyzed changes in board certification status between 2010 and 2012 survey in nine sub cohorts based on their year of registration (defined as those who registered as medical practitioners in or before 1972, then in seven 5-year cohorts from 1973 to 1977 to 2008–2012)(Fig. 1). To examine factors associated with maintenance of board certification, data for the 37,219 physicians who reported that they held board certification in internal medicine were analyzed. We conducted logistic regression analysis to identify factors such as sex, years of experience (0–9, 10–19, 20–29, 30–39, 40–49 and ≥50), workplace (academic hospital, other hospital, clinics or others), type of municipality (city, town or village), and area of practice (internal medicine, subspecialty area in internal medicine) associated with the odds of maintenance of certification. These factors were assessed using 2010 data. Maintenance of board certification was defined as certification in at least one area of internal medicine in both 2010 and 2012 (these could be different areas). Then, status of career transition of board-certified physicians from hospital in 2010 (*n* = 24,788), practice area distribution and board certification status for physicians who moved from hospitals to clinics (*n* = 1063) were also analyzed.

All physicians would report their area of practice, and those who were board certified would report their area of certification; however, some may have been practicing in areas in which they were not board certified. We defined internal medicine physicians as those reported their *main area of practice* as internal medicine, respiratory medicine, cardiology, gastroenterological medicine, nephrology, neurology, diabetes and metabolism, hematology, allergy, rheumatology and/or infectious diseases. Main areas of practice other than internal medicine were classed as subspecialty areas of practice in internal medicine. Thirteen internal medicine subspecialty as for *the area of board certification* were defined with reference to the internal medicine specialty training standards of the Japanese Society of Internal Medicine [27]: general internal medicine, respiratory medicine, cardiology, gastroenterological medicine, hepatology, nephrology, neurology, diabetes, metabolism, hematology, allergy, rheumatology, infectious diseases, and gerontology.

A *P* value of <0.05 was considered statistically significant. IBM SPSS Statistics (version 20.0 J, SPSS IBM Japan Inc., Tokyo, Japan) was used for statistical analysis.

## Results

### Characteristics of study subjects

The majority of respondents in 2010 (59.4%, 61,877 out of 104,193) and 2012 (57.1%, 61,177 out of 107,105) chose internal medicine as their main area of practice, followed by gastroenterological medicine and cardiology. 37.5%, 39,043 out of 104,193 in 2010 and 39.3%, 42,060 out of 107,105 in 2012 internist were board certified in one area or more. Among physicians who reported their area of practice in internal medicine, 84.4% (87,969 out of 104,193) and 83.7%(89, 611 out of 107,105) were men in 2010 and 2012, respectively (Table 1).

**Table 1:** Characteristics of study subjects

		2010 report		2012 report	
		(*n* = 104,193)		(*n* = 107,105)	
Sex (n, %)					
	Male	87,969	84.4	89,611	83.7
	Female	16,224	15.6	17,494	16.3
Years of experience (mean, standard deviation)					
		24.2	14.7	24.5	14.6
Type of municipality (n, %)					
	Special wards	36,303	34.8	38,823	36.2
	Cities	60,871	58.4	61,520	57.4
	Towns and villages	7,019	6.7	6,762	6.3
Type of medical institution (n, %)					
	Academic hospitals	13,717	13.2	14,480	13.5
	Other hospitals	43,967	42.2	45,948	42.9
	Clinics and others	46,509	44.6	46,677	43.6
Main area of practice (n, %)					
	Internal medicine	61,877	59.4	61,177	57.1
	Gastroenterological medicine	12,188	11.7	13,080	12.2
	Cardiology	10,829	10.4	11,541	10.8
	Respiratory medicine	4944	4.7	5337	5.0
	Neurology	4094	3.9	4361	4.1
	Diabetes and metabolism	3488	3.3	3966	3.7
	Nephrology	3085	3.0	3492	3.3
	Hematology	2118	2.0	2353	2.2
	Rheumatology	1058	1.0	1228	1.1
	Infectious diseases	303	0.3	367	0.3
	Allergy	209	0.2	203	0.2
Board-certified physicians (n, %)					
	General internal medicine	13,896	13.3	13,607	12.7
	Gerontology	936	0.9	975	0.9
	Gastroenterological medicine	11,283	10.8	12,090	11.3
	Hepatology	3097	3.0	3583	3.3
	Cardiology	9150	8.8	9884	9.2
	Respiratory medicine	3602	3.5	4027	3.8
	Neurology	3285	3.2	3555	3.3
	Diabetes	3374	3.2	3763	3.5
	Metabolism	1293	1.2	1399	1.3
	Nephrology	2347	2.3	2622	2.4
	Hematology	1876	1.8	2055	1.9
	Rheumatology	1407	1.4	1618	1.5
	Infectious diseases	615	0.6	696	0.6
	Allergy	1196	1.1	1301	1.2
Number of board certifications (n, %)					
	None	65,150	62.5	65,045	60.7
	1	24,149	23.2	26,518	24.8
	2	11,902	11.4	12,381	11.6
	≥ 3	2992	2.8	3161	2.9

### Dynamics of board certification status

Physicians seeking board certification tended to do so earlier in their early career (Table 2). Logistic regression showed that the odds of maintaining board certification were lower in women and those working at locations other than academic hospitals, and higher in those who practiced a subspecialty of internal medicine (Table 3).

**Table 2:** Changes in board certification status between 2010 and 2012

Year of registration as physician	Certified as of 2010 survey	Lost certification	Not reported in 2012 survey	Obtained certification	Not reported in 2010 survey	Certified as of 2012 survey
	(A)	(B)	(C)	(D)	(E)	(F)
2012–2008	0	0	0	0	0	0
2007–2003	890	97	75	2000	163	2881
2002–1998	5183	419	324	1452	388	6280
1997–1993	6579	512	237	860	267	6957
1992–1988	7048	499	228	655	234	7210
1987–1983	6325	523	183	589	151	6359
1982–1978	5539	509	165	536	138	5539
1977–1973	3176	329	122	331	92	3148
1972 or before	4228	955	495	684	189	3651
Total	38,968	3843	1829	7107	1622	42,025

**Table 3:** Factors associated with holding board certification in internal medicine

		Odds ratio	95% Confidence interval			*P* value
Sex						
	Male	1.00	Reference			
	Female	0.87	0.79	–	0.97	0.01
Years since registration as physician						
	0–9	1.00	Reference			
	10–19	1.53	1.32	–	1.77	<0.001
	20–29	1.69	1.46	–	1.96	<0.001
	30–39	1.28	1.10	–	1.50	<0.001
	40–49	0.54	0.45	–	0.64	<0.001
	≥ 50	0.22	0.18	–	0.28	<0.001
Type of municipality						
	City	1.00	Reference			
	Town or village	0.93	0.80	–	1.08	0.31
Type of institution						
	Academic hospital	1.00	Reference			
	Other hospital	0.75	0.67	–	0.84	<0.001
	Clinics and others	0.65	0.57	–	0.74	<0.001
Main area of practice						
	Internal medicine	1.00	Reference			
	Subspecialty area	1.51	1.40	–	1.64	<0.001

### Career transition from hospitals to clinics and practice area

Among hospital physicians with board certification, 4.3% moved to clinics between 2010 and 2012 (Table 4); 87.7% maintained board certification but changed from a subspecialty area to more general internal medicine (Table 5), but 95.8% of physicians who moved to a clinic maintained their practice within internal medicine or one or more of its subspecialty areas (Table 6).

**Table 4:** Status of career transition of board-certified physicians from hospital to clinic

Years since registration as physician	Status in 2010	Status in 2012			
	Hospital physician with board certification	Moved to clinic (*n*)	Moved to clinic (%)	Holding board certification (*n*)	Holding board certification (%)
0–9	2712	115	4.2	97	84.3
10–19	9953	501	5.0	447	89.2
20–29	7551	266	3.5	242	91.0
30–39	3439	121	3.5	99	81.8
40–49	939	50	5.3	40	80.0
≥50	194	10	5.2	7	70.0
Total	24,788	1063	4.3	932	87.7

**Table 5:** Board certification status for physicians who moved from hospitals to clinics between 2010 and 2012

Board certification status		2010 in hospitals		2012 in clinics	
		n	%	n	%
Area of board certification					
	General internal medicine	371	34.9	326	30.7
	Gerontology	27	2.5	21	2.0
	Gastroenterological medicine	302	28.4	270	25.4
	Hepatology	80	7.5	90	8.5
	Cardiology	242	22.8	223	21.0
	Respiratory medicine	116	10.9	103	9.7
	Neurology	70	6.6	60	5.6
	Diabetes	126	11.9	124	11.7
	Metabolism	29	2.7	32	3.0
	Nephrology	87	8.2	79	7.4
	Hematology	36	3.4	30	2.8
	Rheumatology	41	3.9	37	3.5
	Infectious diseases	15	1.4	11	1.0
	Allergy	28	2.6	28	2.6
Number of board certifications					
	0	0	0.0	131	12.3
	1	642	60.4	511	48.1
	2	340	32.0	347	32.6
	≥ 3	81	7.6	74	7.0

**Table 6:** Area of practice and board certification status of physicians who moved from hospitals to clinics between 2010 and 2012 (*n* = 1, 063)

Main area of practice		Internal medicine	Subspecialty in internal medicine	Other area of practice
As of 2010 (Hospital)	Area of practice (%)	334 (31.4)	729 (68.6)	Not applicable
	Number board certified (%)	334 (100)	729 (100)	Not applicable
As of 2012 (Clinics)	Area of practice (%)	611 (57.5)	407 (38.3)	45 (4.2)
	Number board certified (%)	525 (85.9)	377 (92.6)	30 (66.7)

## Discussion

We found that women were less likely to maintain board certification in internal medicine or one of its subspecialty areas after adjusting for possible confounding factors, results that are consistent with our previous study of women in surgery [20]. It appears that women have difficulty maintaining board certification status in internal medicine and surgery. It is therefore critical that obstacles for female physicians must be overcome so as to meet the increasing demand for internists and specialists, for example by creating supportive working environment).

We also found that internal medicine specialists who move to clinics generally stay within internal medicine and maintain board certification, but often change to more general practice. This career pathway is different from that of surgeons, who may change their area of practice from surgery to internal medicine [20]. Japan has well-equipped advanced medical facilities [28], but the number of open hospitals is still limited (918 hospitals out of 8481 facilities as of 2014 [29]). Because surgeons need medical facilities that allow them to perform operations, many who have moved from hospitals to clinics may cease their surgical practice, which likely explains why surgeons who move from hospitals to clinics are less likely to maintain board certification than their internal medicine counterparts. However, this does not mean that board certification requirements differ between types of medical facility. As the role and function of board certification evolves and more physicians seek board certification, the roles of hospitals, clinics and the reimbursement system will also likely to change.

Our results reflect the fact that internists treat large numbers of patients with a wide range of disorders and provide primary medical care and disease management as necessary. In Japan, general practice/family medicine had not been an official category of area of practice and most physicians had been trained in a single area of practice [30, 31]. Therefore, to work in general practice/family medicine, physicians broaden their areas of practice to treat a wider range of patients. The major focus of the current revision of the board certification system to establish a two tier system of basic specialties and subspecialties, and to add general practice as a basic specialty [15]. Currently, the Japanese Society of Internal Medicine has extensively reviewed their board certification system. This will improve the quality of internists, regardless of their area of internal medicine practice.

There will soon be three types of physician working in the area of general practice/family medicine in Japan: those who originally trained with the intention of working in general practice/family medicine; those who trained in internal medicine, worked in a subspecialty and then broadened their area of practice; and those who had been working in areas other than general practice/family medicine and retrained (for example, a surgeon undergoing a career change). As the population ages, the greater demand for disease management will lead to greater demand for generalists, so more emphasis is needed on the training of different types of physicians in general/family practice. Such training must focus on more experienced doctors alike (in terms of continuing education), and those who have different backgrounds and experience.

This study had several limitations. First, the data set did not include data on Board-Certified Members of the Japanese Society of Internal Medicine; this certification is currently a condition for application for most subspecialties as well as application for Fellow of the Japanese Society of Internal Medicine. Second, the Survey of Physicians, Dentists and Pharmacists only establishes whether the respondent is board certified at the time of the survey, and does not collect data on the date of initial certification or its expiry. As one certification is effective for 5 years, the 2-year study observation period could have underestimated the certification maintenance rate. Adding questions to the Survey of Physicians, Dentists and Pharmacists, or establishing an integrated database of physicians’ career characteristics including board certification would overcome this limitation, but this would need the consensus of relevant stakeholders. Third, although the the Survey of Physicians, Dentists and Pharmacists was designed as a census, some individuals did not respond, which might have affected the results. Finally, the main area of practice was self-reported so the actual practice pattern might be different.

## Conclusions

Revision of the board certification system in internal medicine in Japan must consider physicians’ differing career paths, and take into account the needs of female physicians and flexibility to permit mid-career changes. Nevertheless, the quality of certification must be maintained, and the system must allow for sufficient numbers of physicians to be trained and distributed appropriately. It is also important to design programs to train specialists in general practice to meet the increasing demand for general/family practitioners.
